# Transcriptome and DNA Methylation Analyses of the Molecular Mechanisms Underlying with Longissimus dorsi Muscles at Different Stages of Development in the Polled Yak

**DOI:** 10.3390/genes10120970

**Published:** 2019-11-26

**Authors:** Xiaoming Ma, Congjun Jia, Min Chu, Donghai Fu, Qinhui Lei, Xuezhi Ding, Xiaoyun Wu, Xian Guo, Jie Pei, Pengjia Bao, Ping Yan, Chunnian Liang

**Affiliations:** 1 Animal Science Department, Lanzhou Institute of Husbandry and Pharmaceutical Sciences, Chinese Academy of Agricultural Sciences, Lanzhou 730050, China; 82101171175@caas.cn (X.M.); dkjcj@nwafu.edu.cn (C.J.); chumin@caas.cn (M.C.); 82101175147@caas.cn (D.F.); lanlingzi@126.com (Q.L.); dingxuezhi@caas.cn (X.D.); wuxiaoyun@caas.cn (X.W.); guoxian@caas.cn (X.G.); peijie@caas.cn (J.P.); baopengjia@caas.cn (P.B.); 2 Key Laboratory for Yak Genetics, Breeding, and Reproduction Engineering of Gansu Province, Chinese Academy of Agricultural Sciences, Lanzhou 730050, China

**Keywords:** Polled yak, transcriptome, Longissimus dorsi muscle, methylation

## Abstract

DNA methylation modifications are implicated in many biological processes. As the most common epigenetic mechanism DNA methylation also affects muscle growth and development. The majority of previous studies have focused on different varieties of yak, but little is known about the epigenetic regulation mechanisms in different age groups of animals. The development of muscles in the different stages of yak growth remains unclear. In this study, we selected the longissimus dorsi muscle tissue at three different growth stages of the yak, namely, 90-day-old fetuses (group E), six months old (group M), and three years old (group A). Using RNA-Seq transcriptome sequencing and methyl-RAD whole-genome methylation sequencing technology, changes in gene expression levels and DNA methylation status throughout the genome were investigated during the stages of yak development. Each group was represented by three biological replicates. The intersections of expression patterns of 7694 differentially expressed genes (DEGs) were identified (padj < 0.01, |log2FC| > 1.2) at each of the three developmental periods. Time-series expression profile clustering analysis indicated that the DEGs were significantly arranged into eight clusters which could be divided into two classes (padj < 0.05), class I profiles that were downregulated and class II profiles that were upregulated. Based on this cluster analysis, Kyoto Encyclopedia of Genes and Genomes (KEGG) pathway analysis revealed that DEGs from class I profiles were significantly (padj < 0.05) enriched in 21 pathways, the most enriched pathway being the Axon guidance signaling pathway. DEGs from the class II profile were significantly enriched in 58 pathways, the pathway most strongly enriched being Metabolic pathway. After establishing the methylation profiles of the whole genomes, and using two groups of comparisons, the three combinations of groups (M-vs.-E, M-vs.-A, A-vs.-E) were found to have 1344, 822, and 420 genes, respectively, that were differentially methylated at CCGG sites and 2282, 3056, and 537 genes, respectively, at CCWGG sites. The two sets of data were integrated and the negative correlations between DEGs and differentially methylated promoters (DMPs) analyzed, which confirmed that *TMEM8C*, *IGF2*, *CACNA1S* and *MUSTN1* were methylated in the promoter region and that expression of the modified genes was negatively correlated. Interestingly, these four genes, from what was mentioned above, perform vital roles in yak muscle growth and represent a reference for future genomic and epigenomic studies in muscle development, in addition to enabling marker-assisted selection of growth traits.

## 1. Introduction

Yaks (*Bos grunniens*) are large native plateau mammals that have a population of more than 14 million worldwide, principally distributed at high altitudes of 3000 to 5000 m [1]. Domestication of the yak is believed to have occurred during the introduction of farming techniques into the Qinghai-Tibet plateau in the Neolithic Age, approximately 7000–10,000 years ago [2]. Domesticated yaks have since become the most important livestock in the Qinghai-Tibet Plateau and have dispersed throughout the region, following the migration patterns of the Tibetan population [3].

The polled yak is a recent breed developed by the Lanzhou Institute of Animal Husbandry and Veterinary Medicine of the Chinese Academy of Agricultural Sciences, following the development of the Datong yak. High concentrations of protein and unsaturated fatty acids in beef make it nutritious and delicious, but at the same time it has the disadvantages of growing slowly with poor production performance, affecting its monetary and utility values [4]. Therefore, accelerating growth to improve the rate of meat production has become the principal research focus in yak breeding. The molecular genetic mechanisms involved in lengthy development are particularly important. Rates of animal muscle growth and development vary among breeds as important quantitative traits [5,6].

In post-genomic research, transcriptomics is the basis for the interpretation of genomic functional elements and elucidation of the molecular mechanisms of cells and tissues, playing an important role in the research of biological phenotypes and gene expression [7]. In biology a transcript generally refers to an RNA molecule that encodes a protein, and a transcriptome represents the collection of all RNA molecules within a specific organism or tissue within a particular environment, encompassing both coding RNA (mRNA) and non-coding RNA (ncRNA) [8]. Compared with genomes which are relatively stable, transcriptomes change according to physiological state, stage of development, and growth environment [9], having highly dynamic and variable characteristics. The growth and development of skeletal muscle is a complex process, which is promoted through transcriptome regulation involving regulatory networks and signaling pathways. To date, muscle regulatory factors (MRFs) [10], growth hormone (GH) [11], and insulin-like growth factors (IGFs) [12] have been reported to be associated with muscle growth and development. An analysis of the transcriptome of muscle tissue allows the identification of additional candidate genes, regulatory networks, and signaling pathways at the transcriptional level. DNA methylation was the earliest and most studied form of epigenetic DNA molecule modification [13], generally referring to the transfer of a methyl group from S-adenosylmethionine (SAM) as a methyl donor to cytosine under the catalysis of DNA methyltransferase [14]. DNA methylation modification plays a crucial role in mammalian growth and development. It is generally considered to be a type of silent marker that can be stably inherited to the next generation, especially in the inactivation of the X chromosome [15], genomic imprinting [16], and ensuring the stability of transposons in the genome [17]. DNA methylation modifications can be generally divided into three types: promoter CpG island methylation [18], gene body methylation [19], and intergenic region methylation [20]. Generally, DNA methylation is an actively researched area being explored in the context of muscle [21], and DNA methylation is an important step in muscle cell differentiation [22]. In recent years, DNA methylation has become a focus of epigenetic research and now has widespread use in animal husbandry, including a focus on livestock reared for meat and high-value muscle tissue. This type of research focuses on muscle growth and development [23,24].

In summary, DNA methylation is an important epigenetic regulation mechanism, which links the difference between DNA methylation level and gene expression level, and can systematically and comprehensively reveal the epigenetic molecular regulation of differentially expressed genes, because epigenetics can reveal many factors that genomics cannot explain from a deeper and more microscopic perspective. Therefore, MethylRAD and RNA-Seq techniques utilized to study the distribution of methylation and changes to the transcriptome in the longissimus dorsi muscle are meaningful for yak muscle development. Genome-wide DNA methylation patterns in livestock muscle tissue have been previously profiled. For example, Li et al. [25] combined DNA methylation and transcriptome analysis, studying whole genome methylation and gene expression profiles of porcine muscle tissue and the regulatory relationship with microRNA (miRNA), the results of which indicate that DNA methylation status affects gene expression and the manner of transcription affects muscle growth and development [25]. Cao et al. [26] determined a large number of differentially methylated regions (DMRs) and differentially expressed genes based on genome-wide methylation sequencing and transcriptome sequencing technology, finding a number of genes related to the quality of meat in sheep, but no reports have been published concerning the development of yak muscle. In this study, the longissimus dorsi muscle at different stages of development of the polled yak was selected for investigation. Differential methylation and differences in growth and development at different stages were screened. In addition, differentially expressed genes (DEGs), through the combined analysis of methylation and transcriptome data, were explored to reveal the genes involved in the growth and development of skeletal muscle in yaks.

## 2. Materials and Methods

All yaks were handled in strict accordance with good animal practices that comply with the Animal Ethics Procedures and Guidelines of the People’s Republic of China. The present study was approved by the Animal Administration and Ethics Committee of Lanzhou Institute of Husbandry and Pharmaceutical Sciences of the Chinese Academy of Agricultural Sciences (Permit No. 2019-002).

### 2.1. Tissue Collection and Library Preparation

Polled yaks bred via selective breeding were selected from herds in the Ashidan Mountain region of Qinghai Province. The yaks investigated in this study were free grazing within the same pasture, located in Datong County, Qinghai Province. The Datong Breeding Farm in Qinghai Province is located between a latitude of 37°11’~37°32’ north and a longitude of 100°52’~101°26’ east in the southern foothills of Daji Mountain in the Qilian Mountain range. A total of nine healthy female yaks were selected for the study in three age groups, 90-day-old fetuses (group E), six months old (group M), and three years old (group A). Fetal age was estimated from the crown–rump length [27]. Longissimus dorsi muscle samples involved in this study were harvested after slaughter. The samples were dissected into 0.5 cm^3^ cubes and treated with RNAlater (Qiagen, Hilden, Germany) overnight at 4 °C, followed by freezing in liquid nitrogen for RNA extraction, prior to storage at −80 °C. Genomic DNA was isolated using phenol-chloroform extraction. RNA integrity was detected by 1% agarose gel electrophoresis and total RNA concentration and purity obtained by Agilent 2100 assay, RIN, and 28S/18S or 23S/16S.

### 2.2. cDNA Library Construction and Sequencing

First, total RNA was extracted from the samples and the quality of RNA tested. Second, ribosomal RNA from longissimus dorsi muscle tissues was digested using a TruSeq Stranded Total RNA with a Ribo-Zero Gold Kit (Illumina). After removing genomic DNA using DNase I (Fermentas, Vilnius, Lithuania), cDNA was constructed from 2 µg of total RNA per sample following the standard protocol for the Illumina HiSeq 2500 (Illumina Corp, San Diego, CA, USA). Total RNA was purified using Trizol (Invitrogen, Waltham, MA, USA) and quantified using an Agilent 2100 Bioanalyzer (Agilent, Santa Clara, CA, USA). The cDNA libraries were synthesized using mixed mRNA fragments as templates and a TruSeq RNA sample preparation kit V2 (Illumina), in accordance with the manufacturer’s instructions. The libraries were sequenced using an Illumina HiSeq 2500 at Oebiotech, Shanghai, China.

### 2.3. RNA-Seq Data Analysis

Sample paired-end sequencing data were obtained through the Illumina platform. Raw data were then quality checked using the FastQC tool [28]. Per base quality dropped significantly at the 3′-end extremity and a sliding window trimming was performed by Trimmomatic, cutting once the average base quality fell within the Phred score of 10 (90% accuracy) (LEADING: 3 TRAILING: 3 ILLUMINACLIP: TruSeq3-PE-2. fa: 2: 30: 10: 8: true SLIDINGWINDOW: 4: 15 MINLEN: 50) [29]. Trimmed reads were again quality checked using the FastQC tool. The remaining high-quality cleaned reads were aligned to the yak genome (BosGru_v2.0) using HISAT2 [30]. The mRNAs studied in this experiment are based on annotation information of mRNA in the NCBI database (ftp://ftp.ncbi.nlm.nih.gov/genomes/all/GCF/000/298/355/GCF_000298355.1_BosGru_v2.0/GCF_000298355.1_BosGru_v2.0_genomic.gff.gz). Meanwhile, we only utilized the gff information of the protein-encoding RNA for quantification, which automatically filters out the information of the ncRNA. HTSeq-count software [31] was used to obtain the number of reads on the protein-coding genes in each sample and Cufflinks software [32] used to calculate the fragments per kilobase million (FPKM) values in protein-coding genes. Differential expression analysis of the genes was performed using the DESeq package in R software (version 3.5.1). Significantly differentially expressed genes between any two arbitrary samples were identified based on the following thresholds: log 2|fold-change| > 1.2 and padj-value < 0.01 (Benjamini and Hochberg method) [33]. The common expression profiles of the genes among samples were visualized using Venn diagrams (http://bioinformatics.psb.ugent.be/webtools/Venn). Gene ontology (GO) and Kyoto Encyclopedia of Genes and Genomes (KEGG) pathway enrichment analyses were performed using the Bioconductor package clusterProfiler [34]. Ensembl gene IDs were converted into human gene symbols using Biomart (http://www.biomart.org) before conducting enrichment analyses because yak genome annotations are scarce compared with human genome annotations. All obtained *p*-values were adjusted based on the Benjamini and Hochberg method [33].

### 2.4. Time-series Expression Profile Clustering

Time-series expression profile clustering, the nonparametric clustering algorithm of STEM (Short Time-series Expression Miner), version 1.3.11 [35] was used to cluster and visualize expression patterns of DEGs. The maximum unit change in model profiles between time points was adjusted to 2 and the maximum number of model profiles to 20. Expression profiles of genes were clustered based on their log2 (FPKM values) and correlation coefficients. The statistical significance of the number of genes assigned to each profile versus the expected number was computed and corrected for false discovery rate (FDR) at *p* < 0.05 (padj < 0.05) [33].

### 2.5. Quantitative PCR Assay

The differential expression patterns of the genes detected by transcriptome data were validated by qRT-PCR analysis. Nine genes with differences in expression levels related to muscle development and transcriptome sequencing analysis were selected, including *MYOM2*, *ANKRD1*, *MYH7*, *MSTN*, *ACTG1*, *PGAM2*, *LPIN1*, *RXRA*, and *SETD7*. Total RNA (50 μg) was extracted from the nine longissimus dorsi muscle tissues samples using a TRIzol kit (Invitrogen). qPCR was performed using a Roche 480II on samples that had been processed with SYBR Green I. Each qPCR analysis was performed in a 20 μL volume, including 2 μL 1:9 diluted DNA template (<100 ng), 0.4 μL upstream and downstream primers, 10 μL SYBR Premix Ex Taq II reverse transcriptase (10 μM) (TaKaRa, Dalian, China) and 7.2 μL RNase-free ddH2O. The mean number of cycles required to pass the fluorescence threshold (Ct value) of each sample was used to calculate relative gene expression using the 2^−ΔΔ*C*t^ method, with data normalized to the β-actin gene. Primer sequences are listed in Table S1. Three biological repeats were performed for each sample.

### 2.6. MethylRAD Library Sequencing

Genomic DNA of the longissimus dorsi muscle tissues from the three age groups (6 months old, 3 years old, and 90-day-old fetuses) was extracted using the phenol-chloroform extraction method. An aliquot containing 150–200 ng of DNA was used to construct the MethylRAD library, using a previously published protocol [36].

### 2.7. MethylRAD Data Analysis

NGS QC Toolkit software was used to remove adaptors and low-quality reads prior to analysis of MethylRAD data based on a de novo approach and reference genome method. The methylation data of each muscle sample were compared with the reference genome (ftp://ftp.ncbi.nlm.nih.gov/genomes/all/GCF/000/298/355/GCF_000298355.1_BosGru_v2.0/GCF_000298355.1_BosGru_v2.0_genomic.fna.gz) and genome annotation file (ftp://ftp.ncbi.nlm.nih.gov/genomes/all/GCF/000/298/355/GCF_000298355.1_BosGru_v2.0/GCF_000298355.1_BosGru_v2.0_genomic.gff.gz) using SOAP software (ver. 2.21; primary alignment parameters: −r: 0; −M: 4). Untranslated Region (UTR) regions were identified by using SnpEff software (version: 4.1 g) [37] based on an annotation file. The distribution of methylation sites in different gene elements of each sample was plotted using BEDtools software (v2.25.0) [38]. Furthermore, the distributions of the methylated cytosine sites on different elements of the genome, especially on the different regions of genes, were evaluated. The DNA methylation levels of the sites were determined by the reads per million (RPM) value, and the DNA methylation levels of the genes were then evaluated by summing the methylation levels of sites that were localized in the gene regions. The differential DNA methylation levels of sites and genes were identified by using the R package edgeR software [39]. The thresholds were log 2|fold-change| > 1 and *p*-value < 0.05. respectively. Gene ontology (GO) and KEGG pathway enrichment analyses were performed using Bioconductor package clusterProfiler [34], the *p*-values were adjusted based on the Benjamini and Hochberg method [33]. Finally, candidate DMRs in the promoter regions were selected for enrichment and correlation analyses. The DEG and DMP results were correlated using R package ggplot2 [40].

## 3. Results

### 3.1. Alignment to the Reference Genome

In this study, nine cDNA libraries were established, encompassing three different stages of development of the yak (90-day-old fetuses, six months old, and three years old). DEGs related to yak muscle growth and development were identified using RNA-Seq technology. Detailed sequencing and alignment results are shown in Table 1 and Table S2. The nine samples of raw data consisted of more than 97 million DEGs. After mapping to the yak reference genome, the mean total values obtained were 91,129,108, 89,803,981, and 90,960,199, respectively, with mapping ratios of 96.68%, 95.73%, and 95.35%, respectively.

**Figure 1:**
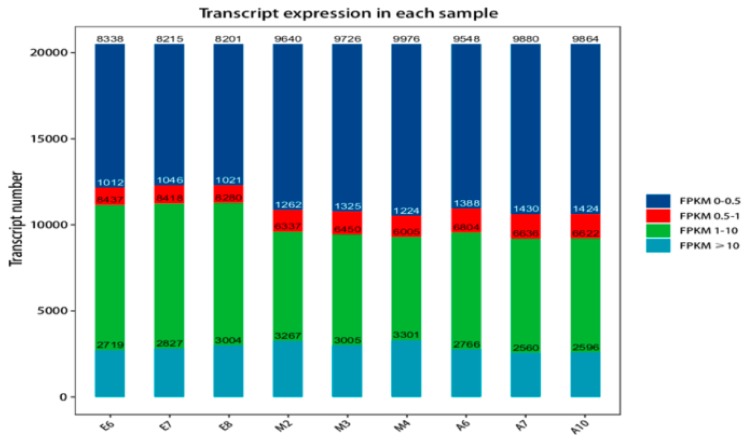
Fragments per kilobase million (FPKM) expression distribution map. The different colors represent different ranges of FPKM values. The abscissa represents different samples and the ordinate is the number of protein-coding genes.

Protein-coding gene expression was calculated as FPKM (fragments per kilobase million) [41], The distribution of the FPKM values of genes is shown in Table S3. Because of differences in the number of genes expressed and degree of gene expression in samples, sample expression value (FPKM) can be divided into different intervals and the numbers of genes expressed over different intervals calculated and displayed as stacked histograms. The distribution of gene expression in the samples is shown in Figure 1.

### 3.2. Differentially Expressed Genes Among the Three Combinations of Groups

**Figure 2:**
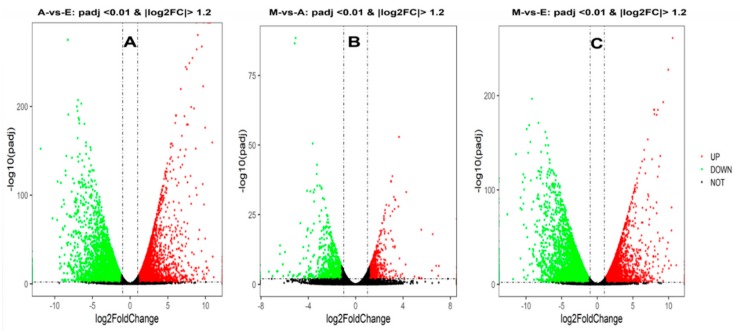
Differential expression volcano map. Differences in gene expression among the 3 combinations of groups, expressed as volcano maps. A black pixel represents a gene where the difference in expression is not significantly different, red and green represent those that are significant; the *X*-axis displays log2 fold change, the *Y*-axis displays the -log10 *p*-value. (**A**–**C**) represents three comparison groups of A-vs.-E, M-vs.-A and M-vs.-E, respectively.

**Figure 3:**
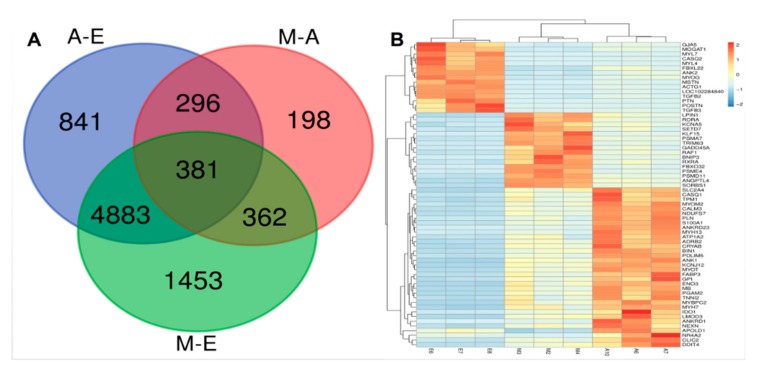
Differentially expressed genes among the three combinations of groups. (**A**) Numbers of expressed and differentially expressed genes: Results of RNA-Seq differentially expressed genes among three comparisons, namely, M-vs.-E, M-vs.-A and A-vs.-E. M, A, and E represent groups of yaks at different stages of development, namely 6-month-old, adult, and fetal, respectively. (**B**) Heat map of differentially expressed genes in pathways related to yak growth for the different gene onology (GO) terms and Kyoto Encyclopedia of Genes and Genomes (KEGG) pathways. Rows indicate genes with significant differences in expression among the three stages; columns represent individual samples from three stages (E, M, and A represent muscle samples from 6 months old, 3 years old and 90-day-old fetuses, respectively). Colors in the figure from red to blue indicate the level of gene expression from high to low.

Analysis of DEGs in the different developmental periods of the longest muscle in the back of the yak was accomplished using DEGseq software. First, *p*-values, padj, and fold-change for all genes in the three comparison groups involved is presented in Table S4. It was found that there were 7079, 1237, and 6401 differentially expressed genes (|log2FC| > 1.2, padj < 0.01) in the 6-month-old vs. fetal yaks (M-vs.-E), 6-month-old vs. adult (M-vs.-A), and adult vs. fetal cattle (A-vs.-E) groups (Figure 2 and Table S4). DEG directional analysis indicated that there were 3096, 3017, and 517 upregulated genes and 3983, 3384, and 720 that were downregulated in A-vs.-E, A-vs.-E, and M-vs.-E comparisons, respectively (Figure S1). In the three comparisons, the number of downregulated genes was higher than the number of upregulated genes. A common cross-analysis found that 381 genes were represented in the intersection of all three groups (Figure 3A), including many genes related to muscle development, e.g., *MYOT*, *LMOD3* and *RXRA*.

Using cluster analysis, comparisons among the three groups indicated that 65 genes were related to yak muscle growth, and based on the data obtained by sequencing, were plotted on a heat map (Figure 3B and Table S5). The different colors in the heat map represent different expression levels, red for high expression and blue for the lowest levels. According to the color distribution in this heat map, the 65 DEGs can be divided into three categories. Compared with the A and M groups, cluster 1 was enriched in group E animals in which a total of 15 genes were significantly upregulated, including *MYL4*, *MSTN*, and *TGFB2*. Genes in the M age group, on the other hand, were upregulated in cluster 2, of which there were 16 DEGs, including *FBXO32*, *TRIM63*, *SETD7*, *LPIN1* and *RXRA*. Compared with the E and M groups, cluster 3 was enriched in group A yaks in which a total of 34 genes were significantly upregulated, including *BIN1*, *MYH7*, and *ANKRD1*. In addition, as shown in the sample clustering results, each of the three samples in each developmental stage was represented in the same cluster in all cases, demonstrating the reliability of the technique for the analysis of muscle samples in the study.

### 3.3. STEM Analysis of DEG Expression Profiles

**Figure 4:**
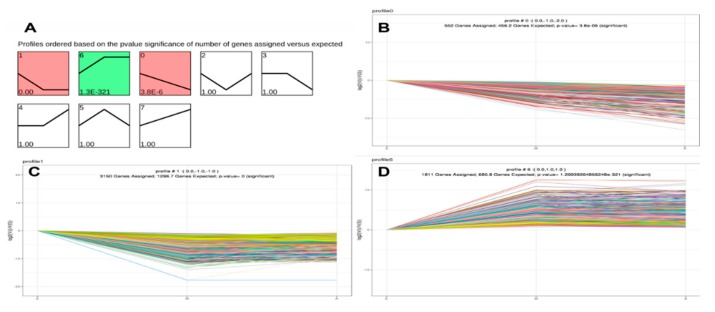
**Short Time-Series Expression Miner** (STEM) analysis of DEG expression profiles. (**A**) Each box corresponds to a type expression profile and only colored profiles are statistically significant. The upper-left and upper-right numbers in each box indicate the order of profiles and *p*-values, respectively. (**B**–**D**) Three significant clusters of DEG profiles across all three stages.

Since the data for this trial were collected from three different time points, we performed clustering of sequence expression profiles to identify the intersection of expression patterns of 7694 genes at the three developmental stages. When the *p*-values (*p* < 0.05) (Figure 4A) were ranked from high to low, a total of three expression patterns were found to be statistically significantly different. A total of 5708 DMGs were clustered into these three profiles, including two downregulation modes (profiles 1 and 0) and one upregulation mode (profile 6) (Figure 4). Thus, the expression patterns of DEGs can be divided into two classes, class I profiles (profile 1 and 0 DEGs) with downregulated patterns and a class II profile (profile 6 DEGs) with upregulated patterns. The results provide new information related to further characterization of novel molecules associated with skeletal muscle development in yaks.

### 3.4. KEGG Enrichment Analysis for Time-series Expression Profile Clustering

**Figure 5:**
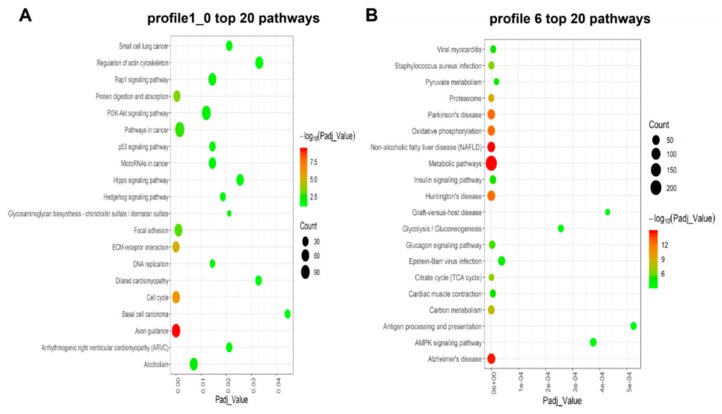
Bubble plot of the top 20 significantly enriched pathways for (**A**) Class I profiles (profiles 0 and 1) and (**B**) Class II profile (profile 6). Colors represent minus logarithms of adjusted padj values. Lengths of columns represent numbers of genes enriched in a pathway.

To further investigate the putative function of DEGs, we performed a KEGG DEG pathway analysis to determine a significant enrichment pathway for each expression profile (padj < 0.05) based on cluster analysis. For the class I profiles (profiles 1 and 0), DEGs significantly enriched 21 pathways (Figure 5A and Table S5), including a number of pathways related to muscle growth and development. These are Focal adhesion, ECM-receptor interaction, PI3K-Akt signaling pathway and Rap1 signaling pathway. Based on the number of DEGs, the three most enriched pathways were Pathways in cancer, PI3K-Akt signaling pathway, and Axon guidance. As for class II profiles, the DEGs were significantly enriched in 58 pathways (Figure 5B and Table S5). The largest number of DEGs and the lowest padj value in the significantly enriched pathway were observed for the Metabolic pathways. Furthermore, Insulin signaling pathway, Cardiac muscle contraction, PPAR signaling pathway, AMPK signaling pathway, and FoxO signaling pathway were also enriched in these profiles.

### 3.5. qPCR Validation of the Three Developmental Stages of Common DEGs

**Figure 6:**
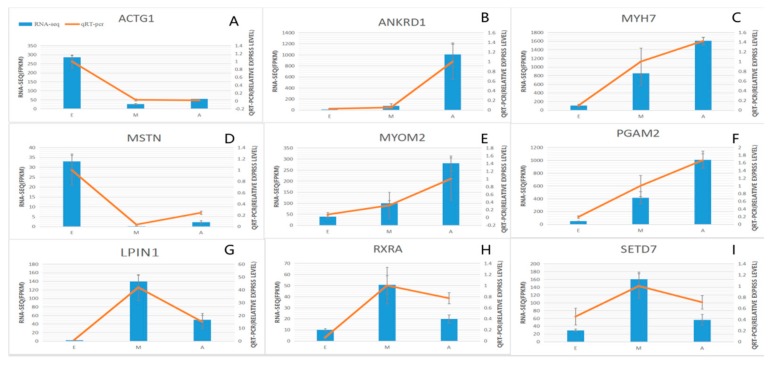
Expression levels of nine DEGs detected by RNA-Seq and validated by qPCR. (A–I) Results from RNA-Seq are shown by bar graphs. Values on the right *Y*-axis represent FPKM. Results of qPCR are shown by line graphs with values on the left *Y*-axis as relative expression levels. Data represent means ± SE.

Samples analyzed by qPCR were the same as those used in RNA-Seq. In this study, 9 differentially expressed genes were selected from 4 significantly enriched growth-related pathways and 2 GO muscle growth-related enrichment entries (Table S7). *MYOM2, ANKRD1, MYH7, MSTN, ACTG1, PGAM2, LPIN1, RXRA* and *SETD7*. The results (Figure 6) demonstrate that the trend in expression of the DEGs in the three groups was consistent with the qPCR results, confirming the reliability of the sequencing data (R = 0.96, *p* = 4.9 × 10^−16^) (Figure S2).

### 3.6. Analysis of DNA Methylation and Its Distribution

The MethylRAD technique was used to analyze cytosine methylation patterns in the longest muscle in hornless yaks at different stages of development (6 months old, 3 years old, and 90-day-old fetuses). In this study, libraries were constructed using MethylRAD technology and sequenced using the Illumina platform. MethylRAD sequencing data (enzyme reads) for each sample were obtained by filtering and deleting labels that did not have the expected restriction site. Changes in the quantity of data before and after data filtering are shown in Table 2. Sequencing depths at DNA methylation sites (CCGG and CCWGG) of each sample are shown in Table S8. In E-group yaks, there were 878,076 and 322,801 instances of methylation at the two types of site, CCGG and CCWGG, respectively. Mean methylation coverage was 34.95% and 21.92%, respectively.

**Figure 7:**
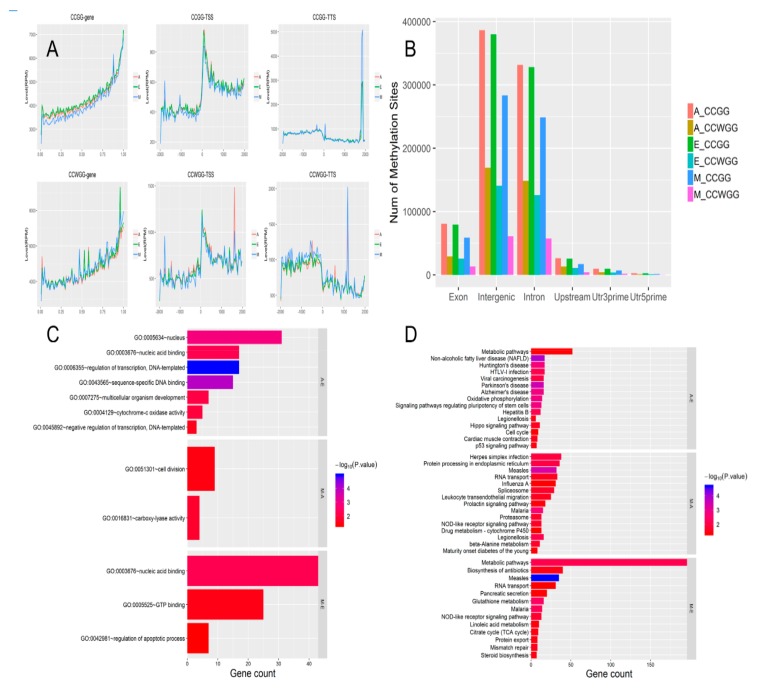
DNA methylation sites, methylation levels, and distribution of functional components. (**A**) Change in DNA methylation levels. In the line chart, red represents the methylation level of the A group; green represents that of the E group and blue represents that of the M group. (**B**) Histogram of distribution of DNA methylation sites in different gene functional components. Exon: exon; Body region: gene area; Intergenic: intergenic region; Intron: intron; Upstream: gene starting sites in a region 2000 bp upstream; Utr3prime: 3’ untranslated region; Utr5prime: 5’ untranslated region. (**C**) Gene ontology (GO) function is classified by histograms of differences in methylation sites. (**D**) Numbers in parenthesis indicate the number of genes affected by differentially methylated regions (DMRs) in a comparison of different groups. Colors represent minus logarithms of adjusted *p*-values. Lengths of columns represent numbers of genes enriched in a pathway.

A total of 661,244 CCGG and 145,288 CCWGG DNA methylation sites were found in the M group, with mean methylation coverage of 13.49% and 11.63%, respectively. Similarly, a total of 891,911 CCGG and 383,979 CCWGG DNA methylation sites were found in Group A, with mean methylation coverage of 30.11% and 17.74%. The degree of methylation in group M was smaller than that of the other two groups. We used the wild-type yak genome to annotate measured DNA methylation data and mapped the sequences centered on the transcription start site (TSS) represented by the upstream TSS of DNA methylation and the sequence downstream of the transcription center represented by the TSS (Figure 7A). The data indicate that the methylation sites on DNA were principally distributed within the intergenic regions and introns. It can be seen that the degree of methylation in the M group was lower than in the other two groups (Figure 7A).

### 3.7. Differential Methylation Regions Among the Three Groups

**Figure 8:**
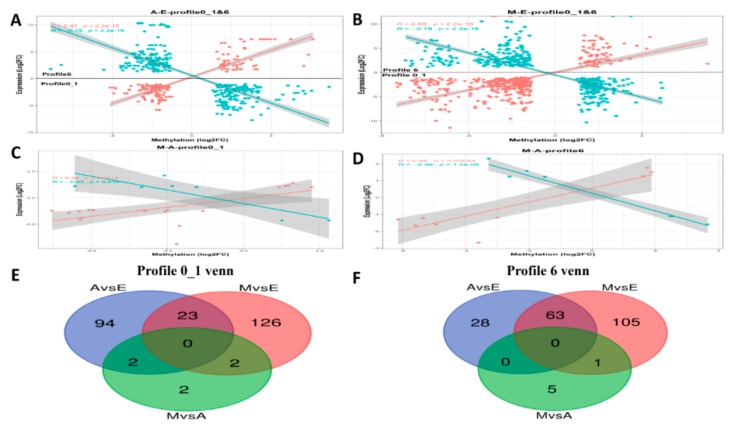
Pearson correlations of fold-change in gene expression and differential methylation rates for DEGs in muscle tissues and Venn diagrams of numbers of differentially methylated promoters (DMPs) among different comparisons. Scatter plots for (**A**) A-E, (**B**) M-E and (**C**,**D**) M-A. The *X*-axis represents mean difference in methylation ratio between M and E groups; the *Y*-axis represents the logarithm of fold-change (log 2 FC). The blue dots represent negatively correlated genes and red dots represent positively correlated genes. The trend lines and formula in each scatter plot represent the correlation coefficients.

Based on the sequencing depth information for each locus in each sample, the *p*-values of the differences and multiple (Log2FC) of differences for each locus between groups were calculated using edgeR software [39]. When comparing groups, a more reliable method was used, as follows: for a site where a statistical comparison was calculated, at least one group was required to have all samples within that group possessing a sequencing depth greater than 3 at that site. Only sites with a *p*-value ≤0.05 with an absolute Log2FC value greater than 1 were considered differential sites and categorized as either upregulation or downregulation sites as appropriate. We obtained the DNA methylation site annotation of the yak genome, and found that the DNA methylation site distribution curve had TSS representing an upstream sequence centered on the transcription initiation site, and TTS representing a downstream sequence centered on the transcription termination site (Figure 8A). The three combinations of comparison (M-vs.-E, M-vs.-A, A-vs.-E) had 1344, 822, and 420 differential genes with CCGG sites, respectively (Figure 7B), and 2282, 3056, and 537 that had CCWGG sites, respectively (Figure 7B).

### 3.8. Enrichment Analysis of DMRs

The differentially methylated regions (DMRs) obtained above were explored by GO and KEGG analyses to reveal functional enrichment in each group. GO enrichment analysis identified that nucleic acid binding, regulation of apoptotic process, GTP binding, and carboxy-lyase activity were significantly enriched. A number of these pathways are associated with muscle growth and development (Figure 7C). In the KEGG analysis, 15 significant pathways were identified when the three developmental stages were compared (Figure 7D) (M-vs.-E, M-vs.-A, A-vs.-E). Of these, Hippo signaling pathway, p53 signaling pathway, and steroid biosynthesis are closely related to the growth and development of skeletal muscle.

### 3.9. Integrated Analysis of DEG and DMP Results

Of the methylated regions, methylation of the promoter regions is most important [42], so in this study we conducted differential analysis of these regions of greatest interest, denoting them differentially methylated promoter (DMP) regions. Although the DEGs co-expressed in the time-series expression profile clustering analysis, the mechanisms by which DEGs and DMPs affect the growth of the yak dorsal muscles were unclear. First, we performed transcriptome analysis of the results with an overlap of statistics of the DMPs in the three comparison groups (Table 3 and Table S9). Subsequently, linear relationship analysis was performed based on the results above. It was necessary to conduct a correlation analysis of genes shared between the DEGs and DMPs. We focused on the correlation between muscle DEG log2(FC) values and differences in DMP gene methylation. Additionally, the two sets of data were integrated and the results of negative correlation between DEGs and DMPs analyzed (Figure 8A–D). Figure 8A–B contains two types of profile (profile0_1 and profile6), which have been brought together in one image to better demonstrate the linear relationship between DMPs and DEGs, because the two types of significant profiles in the time-series expression profile were compared in the E group with the other two groups (Group E up- or downregulated compared with the other two groups), while M-vs.-E and A-vs.-E in the comparative analysis of DMPs also exhibited the same trend. Figure 8 includes Venn diagrams of the negative correlation results above of the three developmental stages in profile1_0 (Figure 8E) and profile6 (Figure 8F). In order to find more evidence supporting the correlation between gene expression and genome methylation, the two sets of data between the two groups were analyzed for subsequent analysis (Table 4).

## 4. Discussion

In recent years, a large number of studies have shown that DNA methylation has a complex regulatory effect on the expression levels of genes. However, investigations of the mechanisms of the regulation of DNA methylation at a gene transcription level remain at the exploration stage [43]. Combined with an extensive reference of second-generation sequencing in animal husbandry, considerable second-generation sequencing and omics analyses have been conducted in cattle, pigs, chicken, and other species to ascertain which genes are related to muscle growth or affect meat quality. Although DNA methylation has been extensively studied, few studies have focused on the yak, a plateau endemic species. This study provides a comprehensive analysis of skeletal muscle DNA methylation patterns in the different stages of development of the yak. The purpose of this study was to find methylated genes that affect yak skeletal muscle development.

### 4.1. KEGG Analysis Based on Time-series Expression Profile Clustering

Since our experimental data is derived from different developmental periods, we utilized STEM software to analyze the data and apply them to the process of dynamic biology [44,45]. We identified important time-expression profiles associated with these expression profiles, retaining the important profiles which could then be combined into clusters for further data mining [35]. In the present study, three significant expression profiles were identified and combined into two clusters, class I profiles (profiles 1 and 0) with downregulated patterns and a class II profile (profile 6) with an upregulated pattern. According to the results of the STEM software analysis, it was found that there were cases in which genes were downregulated in the class I profiles, for example, *PAX7, Col4A1, Col4A2, IGF2R, IGF2, FN1, MYL4*, and *MYL7*, consistent with research on cattle and goats [46,47]. In the class II profile, many upregulated genes were observed, including *LMOD2, MUSTN1, XIRP2, PDLIM5, MYF6*, and *TNNT1*, also in accordance with a previous report on cows [47]. For instance, many MYL protein family members were found in this study. MYL7 cardiac muscle light chain polypeptide protein 7 exhibits an inhibitory effect on the differentiation of cardiomyocytes and has a role in regulating the actin cytoskeleton. The *MYL4* gene encodes the alkaline light chain of myostatin, an important component of the complex that constitutes muscle myosin, principally expressed in the atrium of embryonic muscle and adult animals [48]. It is related to the formation of embryonic muscle structure, muscle development, muscle contraction, and myosin formation [49]. In the present study, it was found that *MYL4* and *MYL7* were only expressed in fetuses, consistent with previous studies, indicating that the two genes play a regulatory role in the development of muscle during the fetal period.

After analysis of the expression levels of the transcriptome of the longissimus dorsal muscle tissue of nine yaks from three stages of development, KEGG enrichment analysis of the differential genes in the class I and II profiles was conducted. In class I profiles, it was found that many of the pathways were related to muscle growth, intermuscular fat deposition, and energy metabolism, namely the PI3K-Akt signaling pathway, Rap1 signaling pathway, focal adhesion, and ECM-receptor interaction. It has been demonstrated that myoblast differentiation is mediated through the PI3K-Akt signaling pathway [50]. Studies have shown that ucOC induces myoblast proliferation through sequential activation of the PI3K-Akt and p38 MAPK pathways in C2C12 myoblasts [51]. When stimulated by insulin-like growth factor (IGF), PI3K-Akt signaling positively regulates myogenic differentiation [50]. It has been reported that inhibition of PI3 kinase or its downstream target Akt prevents muscle differentiation in cell culture, while expression of active PI3 kinase and Akt induces myogenic differentiation [52]. Interestingly, Rap1 signaling interacts with the β-adrenergic signaling pathway and has been shown to play a key role in skeletal muscle growth and development [53]. Previous studies have shown that Rap1 proteins accumulate in specialized muscle cell domains and undergo important modifications in the early and late stages of myogenesis: they are neuromuscular and tendon junctions [54]. Therefore, it was found that the majority of genes in the cluster were downregulated, suggesting that these genes are involved in the development of muscles and dependent on satellite cell formation. In short, these DEGs in the downregulated pattern were significantly enriched in the corresponding pathways, suggesting that they play a role in the early development of fetal muscle.

Within the class II profile, three pathways related to growth were identified, namely, metabolic pathway, insulin signaling pathway, and regulation of the actin cytoskeleton. Transcriptome KEGG cluster analysis showed that the metabolic pathway’s padj value was the smallest (Figure 5B). Previous studies have shown an increase in gene expression involved in the energy metabolism pathway. The high levels and upregulation of creatine kinase (CKM) in muscle also support an increase in energy levels, which is associated with hypertrophic growth of muscle [55]. Interestingly, we also found many pathways for glucose metabolism in these pathways, including the insulin signaling pathway, AMPK signaling pathway, and glycolysis/gluconeogenesis. Skeletal muscle is the main site of glucose storage and metabolism, and the secretion of insulin and contraction of muscle stimulates the transport of glucose transporter (GLUT4) from the cytoplasm to the cell membrane, stimulated through different signaling pathways [56]. AMP-activated protein kinase (AMPK) is a key factor in skeletal muscle contraction and exercise-mediated glucose uptake [57,58]. It is well known that the PPAR pathway is expressed not only in fat but also in skeletal muscle [59]. In addition, the PPAR signaling pathway is also enriched in these profiles, as determined by KEGG analysis. Insulin is an important factor in energy conversion, playing a crucial role in the development of muscle. Studies have shown that the expression of PPARγ in skeletal muscle assists in improving insulin sensitivity [60], including *RXRA* and *ANGPTL4* in the PPAR pathway. The *ACSL4* gene is involved in the metabolism of lipids and carbohydrates [61]. From research in livestock, the *ANGPTL4* gene has been exploited as a candidate gene for intermuscular fat deposition and a marble phenotype in meat [62], but research on the metabolism of lipid in yak muscle has not yet been conducted. In short, the DEGs which correspond to significantly enriched pathways in the class II profile with upregulated patterns have revealed the role of these genes in the late stages of yak muscle development.

### 4.2. Longissimus Dorsi Muscle Methylation Profile and Correlation Analysis of DMR Signaling Pathway

It was found that the whole genome methylation profile of yak muscle tissue and methylation modification distribution of expression level genes and nearby regions in all samples were similar to those of other species [63,64] (Figure 7A). The degree of methylation was similar at the three developmental stages of the yak, with the number of CCGG methylation sites higher than CCWGG sites (Figure 7B). Analysis of pathways and genes associated with DMRs revealed that hypermethylated genes were significantly concentrated in three respects: a pairwise comparison of data from three stages revealed that pathways for muscle growth and development existed in the A-vs.-E and M-vs.-E groups, the Hippo signaling pathway, and p53 signaling pathway in the A-vs.-E group and steroid biosynthesis in the M-vs.-E group (Figure 7D).

Our study found two key genes involved in the Hippo signaling pathway: *Wnt10b* and *Fzd7*. It has been reported that *Wnt10b*, a ligand gene of the Wnt ligand gene family, specifically activates typical Wnt/β-catenin signaling, thereby activating β-catenin/LEF/TCF-mediated transcription [65]. *Wnt10b* also plays an important role in muscle development [66]. Jeong et al. used fluorescence quantification to demonstrate that the Wnt10a/β-catenin pathway inhibits IMF (intramuscular fat) deposition in the longissimus dorsi muscle of Korean cattle [67]. *Fzd7* acts as a receptor for *Wnt7a*, and the signaling pathway comprising the two directly activates myogenic lineage progression at different stages [68]. In addition, Júnior et al. used the BovineHD chip to correlate the carcass traits of Nellore cattle, finding that the REA (Ribeye area) trait was significantly associated with the bta8 (Bos taurus chromosome) 107978188bp-108270826bp region in cattle, which includes the *CDKN2A* gene [69]. In the comparison of 6-month-old with fetal (M-vs.-E) group the longissimus dorsi muscle methylation differential gene analysis, the steroid biosynthesis pathway was significantly enriched. SOAT1 (sterol O-acyltransferase 1) acts as an enzyme encoding a network involved in steroid and lipogenesis/fat decomposition, and has been used as a candidate gene for bovine growth traits [70].

### 4.3. Negative Correlation Between Methylation Levels of DMPs and Differential Expression of DEGs

There is a complex relationship between trends in gene expression and gene methylation modifications. Studies have shown that methylation of the promoter region blocks the occurrence of transcription, while the process of methylation modification in other regions remains unclear [43]. In summary, this study demonstrated an epigenetic effect in addition to genomic variation through analysis of a significant linear correlation between gene expression and methylation modification.

In addition to a linear analysis of the two sets of data, in order to better locate the muscle development genes and plot DEG and DMP Venn diagrams of the three stages of development, it was found that several key genes in the M-vs.-E and A-vs.-E comparisons that affect muscle development and fat deposition, as plotted in the Venn diagram, include insulin-like growth factor 2 (IGF2), transmembrane protein 8c (TMEM8C, also called myomaker), musculoskeletal embryonic nuclear protein I (MUSTN1) and calcium voltage-gated channel subunit alpha1 S (CACNA1S).

Within the class I profile (profile 0_1), according to the Venn diagram (Figure 8E), at the intersection of the M-vs.-E and A-vs.-E groups, the two key genes are *IGF2* and *TMEM8C*, respectively. *IGF2* plays an important role in growth and development in animals. Numerous studies have demonstrated that *IGF2* exerts precise regulatory control on skeletal muscle growth and development, muscle remodeling, and hypertrophy, functions that require the activation and differentiation of muscle satellite cells. The *IGF2* gene is also among the earliest identified imprinted genes. Paternal expression and maternal imprinting are important in cell proliferation, differentiation, apoptosis, and transformation [71]. Studies have shown that the locus for *IGF2* is at the end of the short arm of chromosome 2 in pigs, a key quantitative trait locus (QTL) affecting muscle growth and fat deposition [72]. The composition and number of muscle fibers in porcine skeletal muscle play a decisive role in pork quality and yield, while the number of muscle fibers is determined during the prenatal embryonic period. Genome-wide transcriptome expression profiling of fetal skeletal muscle in Chinese local breed Tongcheng pigs and the foreign breed Changbai pig after 33, 65, and 90 days of pregnancy indicated that the *IGF2* gene is differentially expressed by reference to a transcriptome expression profiling library [73]. *IGF2* can promote the precipitation of fat. When the concentration of *IGF2* in plasma increases, body weight also increases accordingly, in addition to an increase in mass of the long back muscle, thickness also increasing significantly [74]. The expression of the *IGF2* gene was found to be higher in the fetal yak than in the other two groups. It was also found that methylation in the promoter region was higher in the fetal yak group than in the other two groups. The results were consistent with those found by Huang and colleagues in cattle [75]. Zhang et al. used an *IGF2* overexpression vector transfected into yak fibroblasts and found that *IGF2* has a negative regulation mechanism on the *PIK3CG* gene in the PI3K-Akt signaling pathway in yak cells, thereby regulating fibroblast proliferation that affects the growth of individual animals [76]. In addition, TMEM8C is a member of the TMEM protein family. Millay et al. [77] identified a muscle-specific transmembrane protein and named it myomaker (TMEM8C), which identified TEME8C as being directly involved in the process of myoblast fusion, promoting the fusion of myoblasts. In the study of bovine species, Miretti et al. [78] found the gene expression patterns of myoblasts during adult bovine satellite cell differentiation into myoblasts, demonstrating that myoblasts may be the basis of bovine myoblast formation. In recent years, Huang et al. [79] found that the expression of the myomaker gene was negatively correlated with the degree of methylation in its promoter region during postnatal development in the muscle tissue of the Japanese flounder, suggesting that DNA methylation patterns in the myomaker promoter were involved in controlling gene expression. However, the role of its expression patterns and methylation of the promoter in the yak has not yet been studied. In the present study, we found that the expression of myomaker in the embryonic stage was higher than in the other two periods, and that methylation of the promoter region in the A and M groups occurred. This result is consistent with those found by Huang and colleagues in the Japanese flounder [79], suggesting that myomaker regulates muscle formation during muscle development.

Within the class II profile (profile 6), According to the Venn diagram (Figure 8E), at the intersection of the M-vs.-E and A-vs.-E groups, the two key genes are *CACNA1S* and *MUSTN1*, respectively. *CACNA1S* is the most important member of skeletal muscle encoding the L1 VGCCs (voltage-gated calcium channels), channel a1 subunit gene, also known as Ca_v_1.1. The expression of *CACNA1S* is regulated by a variety of factors, including aging [80,81], development [82], and muscle denervation [83], in addition to insulin-like growth factors and age [84], all of which affect the expression of *CACNA1S*. At present, the *CACNA1S* gene is less studied in livestock breeding, mostly concentrated on pig research. Fronieke et al. [84] proposed that the homologous sequence of the human *CACNA1S* gene is present on pig chromosome 9. Fang et al. [85] speculated that it is a suitable candidate gene for being a QTL affecting pork quality traits. They located the porcine *CACNA1S* gene in a radiation hybrid cell line for the first time, at 10pl1-12. In addition, The *MUSTN1* gene encodes a total of 82 amino acids and was first discovered and validated in mice in 2004 [86]. There are six AP-1 transcription factor binding sites in the promoter region of this gene. It was found that two members of one family of AP-1 transcription factors, c-Fos and FRA2, are responsible for the transcriptional activity of this gene, a key factor in skeletal muscle-specific regulation [87]. Therefore, we hypothesize that *MUSTN1* is a skeletal muscle-specific regulatory gene. In this study, the expression level of the *MUSTN1* gene was least during the embryonic stage and the difference between the other two stages was not significantly different. The degree of methylation in the promoter region was essentially the same as that in group A. These observations were similar to observations in sheep [88], in which Gao et al. analyzed the longissimus dorsi muscles at different stages of development. Analysis by qPCR indicated that gene expression was lowest in newborn animals. Furthermore, differential expression in tissues may be related to animal muscle growth rate and tissue specificity, the speed of animal muscle growth being modulated by genetics [89], gender [90], and the uterine environment [91]. After the integration of two types of analyses, the important genes identified in this study are summarized in Table 4, which provides a full view of genes supported by multiple evidences. Our results indicate that muscle development-related genes are associated with the growth hormone and metabolism of muscle cells. We believe that these genes might partially contribute to the yak growth difference in three growth stages. However, the epigenetic effects of these genes on yak growth still require further study in the future.

## 5. Conclusions

In the present study, we systematically identified DEGs associated with yak skeletal muscle development and investigated their temporal expression profiles during skeletal muscle development across three developmental stages using RNA-Seq. Functional enrichment analysis of DEGs suggests that biological pathways are directly linked to the temporal changes during yak skeletal muscle development. Additionally, high-resolution DNA methylation profiles of the whole genome from longissimus dorsi muscle tissues were analyzed for three different growth stages of the yak. Combined with the results of time-series expression profile clustering analysis, we studied the regulation of DNA methylation at the transcriptome level by promoters and confirmed many important genes related to DMPs. We identified a number of striking DNA methylation changes, such as a tendency toward methylation in gene promoters in the longissimus dorsi muscles of the yak. Furthermore, we identified numerous genes that exhibited age-related methylation changes and are potentially involved in the aging process. Several genes were highlighted due to their known association with muscle development, including *CACNA1S, IGF2, MUSTN1,* and *TMEM8C*. We identified differences in the expression and differential promoter methylation of four genes, which may affect the development of yak muscles and the quality of its meat. In future studies, the function of these genes will require investigation to a greater depth. Our findings provide valuable data for a more detailed understanding of the genetic and epigenetic mechanisms that control the economic traits of cattle and can be used to label assisted selection programs to improve yak meat production.

## Figures and Tables

**Table 1:** Sequencing data quality preprocessing results.

Sample	Raw_Reads	Raw_Bases	Clean_Reads	Clean_Bases	Valid_Bases	Q30 ^1^	GC
E6	98.03M	14.70G	93.46M	13.67G	92.96%	94.20%	46.73%
E7	98.27M	14.74G	94.05M	13.77G	93.45%	94.57%	47.06%
E8	99.04M	14.86G	93.91M	13.73G	92.43%	93.74%	47.61%
M2	97.85M	14.68G	95.03M	13.67G	93.16%	94.91%	49.89%
M3	98.12M	14.72G	95.08M	13.78G	93.64%	95.01%	48.90%
M4	99.61M	14.94G	96.08M	13.86G	92.75%	94.70%	51.32%
A6	98.22M	14.73G	93.56M	13.68G	92.87%	94.33%	48.93%
A7	99.56M	14.93G	94.64M	13.86G	92.83%	94.00%	47.78%
A10	99.28M	14.89G	94.57M	13.86G	93.07%	94.27%	48.58%

^1^ Q30: Bases with a Phred values greater than 30 in Raw_Bases as a percentage of the total number of bases.

**Table 2:** Sample sequencing data volume and comparison rate.

Sample	Raw Reads	Enzyme Reads	Mapping Reads	Ratio
E6	139,680,224	64,643,466	30,210,658	46.73%
E7	142,659,082	65,566,251	38,487,733	58.70%
E8	142,659,082	75,344,977	35,403,055	46.99%
M2	36,447,959	21,083,527	9,529,418	45.20%
M3	36,447,959	22,576,370	10,604,071	46.97%
M4	36,447,959	23,206,057	11,126,778	47.95%
A6	139,680,224	57,187,481	30,914,358	54.06%
A7	139,680,224	65,584,779	30,544,305	46.57%
A10	139,680,224	65,816,098	32,854,859	49.92%
Average	105,931,437	51,223,223	25,519,471	49.23%

**Table 3:** Statistics of two sets of data overlap.

Profile	Profile Number	DMP Group	Two Set Data Overlap Number of DMPs
profile1_0	1811	A-vs.-E	274
profile1_0	1811	M-vs.-A	27
profile1_0	1811	M-vs.-E	574
profile6	3702	A-vs.-E	214
profile6	3702	M-vs.-A	18
profile6	3702	M-vs.-E	323

**Table 4:** Muscle developmental genes shared between different groups according to DMPs and DEGs.

Gene	Description	Group
*IGF2*	insulin-like growth factor 2	M-E; A-E; profile0_1
*TMEM8C*	transmembrane protein 8c	M-E; A-E; profile0_1
*MUSTN1*	musculoskeletal, embryonic nuclear protein I	M-E; A-E; profile6
*CACNA1S*	calcium voltage-gated channel subunit alpha1 S	M-E; A-E; profile6

## Supplementary Materials

The following are available online at https://www.mdpi.com/2073-4425/10/12/970/s1, Table S1: Primers used for the validation of differentially expressed genes (DEGs) by qRT-PCR. Table S2: Reads and reference genome comparison rate statistics. Table S3. Distribution of fragments per kilobase million (FPKM) values of genes. Table S4: Total genes and the different expression genes in muscles of yaks. Table S5: The FPKM values of 65 genes from 9 samples used in the heat map (Figure 3B). Table S6. Significantly enriched Kyoto Encyclopedia of Genes and Genomes (KEGG) pathways for Class I profiles and Class II profiles. Table S7 KEGG pathway and gene ontology (GO) terms associated with muscle development. Table S8: Depth of longissimus dorsi muscle methylation at different stages of yak development. Table S9: Gene list of integration of DEG and differentially methylated promoter (DMP) results. Figure S1: Numbers of upregulated and downregulated genes in yak longissimus dorsi muscles through pairwise comparisons. Figure S2: Correlation values between the qPCR and the RNA-Seq data. The trend lines and formula in each scatter plot represent the correlation coefficients.

## Abbreviations

ACTG1: actin γ 1, Adssl1: adenylosuccinate synthetase like 1, ANKRD1: ankyrin repeat domain 1, ANKRD37: ankyrin repeat domain 37, BIN1: bridging integrator 1, CACNA1S: calcium voltage-gated channel subunit alpha1 S, COL16A1: collagen type XVI α 1 chain, COL4A1: collagen type IV α 1 chain, COL4A2: collagen type IV α 2 chain, DEGs: differentially expressed genes, DMP: differentially methylated promoter, DMRs: differentially methylated regions, DUSP1: dual specificity phosphatase 1, FBXO32: F-box protein 32, FN1: fibronectin 1, FPKM: fragments per kilobase million, GH: growth hormone, GO: Gene ontology, IGF: insulin-like growth factors, IGF2: insulin like growth factor 2, IGF2R: insulin like growth factor 2 receptor, KEGG: Kyoto Encyclopedia of Genes and Genomes, LMOD1: leiomodin 1, LMOD2: leiomodin 2, LMOD3: leiomodin 3, LPIN1: lipin 1, mRNA: message RNA, MSTN: myostatin, MUSTN1: musculoskeletal, embryonic nuclear protein I, MYF6: myogenic factor 6, MYH7: myosin heavy chain 7, MYL4: myosin light chain, MYL7: myosin light chain 7, MYOM2: myomesin 2, MYOT: myotilin, ncRNA: Non-coding RNA, NQO1:NAD(P)H quinone dehydrogenase 1, PAX7: paired box 7, PDLIM5: PDZ and LIM domain 5, PGAM2: phosphoglycerate mutase 2, RAP1: DNA-binding transcription factor, RXRA: retinoid X receptor α, SAM: S-adenosylmethionine, SETD7: SET domain containing 7, SNED1: sushi, nidogen and EGF like domains 1, STEM: Short Time-series Expression Miner, TGFB2: transforming growth factor β 2, TMEM8C: transmembrane protein 8c, TNNT1: troponin T1, slow skeletal type, TRIM63: tripartite motif containing 63, TSS: transcription start site, UTR: Untranslated Region, XIRP2: xin actin binding repeat containing 2.
