# Supplementary material for: Transcriptome and DNA Methylation Analyses of the Molecular Mechanisms Underlying with Longissimus dorsi Muscles at Different Stages of Development in the Polled Yak
Source: Genes (Basel). 2019 Nov 26;10(12):970. doi: 10.3390/genes10120970 (PMC6947547; doi:10.3390/genes10120970)
Supplement: Supplementary file 1 [file genes-10-00970-s001.zip › Table S7.docx]

| **Term** | **Count** | **PValue** | **Genes** |
| --- | --- | --- | --- |
| **FoxO signaling pathway** | 11 | 0.000463 | SLC2A4, TGFB3, RAF1, SETD7, BNIP3, FBXO32, IL7R, PIK3R3, GADD45A, CCNG2, TGFB2 |
| **Metabolic pathways** | 41 | 0.075505 | ETNPPL, ACOX2, NAMPT, SEPHS2, PGAM2, ALG8, UXS1, CMBL, ALDH1A1, ST3GAL1, NDUFS7, LOC102286314, ALAS1, LOC102283235, TPI1, B3GALNT1, ALDH1A3, MGLL, ENO3, ACSL4, AGPAT2, DHCR24, PLD3, ADSSL1, MSMO1, MAOB, PNPLA2, DLAT, IDO1, ACACB, LPIN1, AMPD3, TST, PYCR1, LOC102274962, GPI, GLUL, AHCYL2, GPAM, ACSM5, REV3L |
| **Tight junction** | 8 | 0.00233 | ACTG1, LOC102284840, YBX3, LOC102284275, MYH13, LOC102283996, LOC102284556, LOC102279539 |
| **Adrenergic signaling in cardiomyocytes** | 9 | 0.01114 | ADRB2, MYL4, PLN, CREM, CALM3, MYH7, ATP1A2, CACNA2D2, TPM1 |
| **PPAR signaling pathway** | 6 | 0.01888 | ACOX2, SORBS1, RXRA, FABP3, ACSL4, ANGPTL4 |
| **GO:0003012 muscle system process** | 30 | 2.0E-10 | PLN，MYOM2，KCNA5，CASQ2，MYH13，CASQ1，KCNJ12，MYBPC2，PDLIM5，MYL4，TNNI2，ADRB2，SORBS1，MSTN，CLIC2，TRIM63，CRYAB，MYOT，ANK2，KLF15，LMOD3，MYOG，PGAM2，GJA5，TPM1，ATP1A2，MB，MYL7，MYH7，CALM3 |
| **GO:0042692**  **muscle cell differentiation** | 25 | 4.27E-9 | MYF5，ABL1，MAMSTR，IFRD1，MYOM2，CASQ2，POPDC3，BARX2，RBM38，CASQ1，ACTG1，MYBPC2，PDLIM5，HES1，RXRA，ANK2，LMOD3，SORT1，BIN1，CACNA2D2，MYOG，TPM1，RORA，NEXN，ANKRD1 |
