# Supplementary material for: Transcriptome and DNA Methylation Analyses of the Molecular Mechanisms Underlying with Longissimus dorsi Muscles at Different Stages of Development in the Polled Yak
Source: Genes (Basel). 2019 Nov 26;10(12):970. doi: 10.3390/genes10120970 (PMC6947547; doi:10.3390/genes10120970)
Supplement: Supplementary file 1 [file genes-10-00970-s001.zip › Table S8.docx]

**Table S6.** Depth of *longissimus dorsi* muscle methylation at different stages of yak development.

| **Sample** | **CCGG** | | | **CCWGG** | | |
| --- | --- | --- | --- | --- | --- | --- |
|  | **Number of Sites** | | **Depth** | **Number of Sites** | | **Depth** |
| M2 | 603169 | 13.21 | | 129978 | 10.68 | |
| M3 | 694705 | 13.13 | | 151523 | 11.53 | |
| M4 | 685859 | 14.13 | | 154363 | 12.69 | |
| A6 | 917271 | 29.14 | | 385214 | 18.24 | |
| A7 | 824591 | 31.23 | | 363260 | 16.99 | |
| A10 | 933871 | 29.97 | | 403464 | 17.98 | |
| E6 | 866202 | 29.5 | | 360248 | 17.99 | |
| E7 | 930431 | 37.73 | | 314910 | 25.98 | |
| E8 | 837594 | 37.62 | | 293244 | 21.79 | |
