# Supplementary figures and images for: Transcriptome and DNA Methylation Analyses of the Molecular Mechanisms Underlying with Longissimus dorsi Muscles at Different Stages of Development in the Polled Yak
Source: Genes (Basel). 2019 Nov 26;10(12):970. doi: 10.3390/genes10120970 (PMC6947547; doi:10.3390/genes10120970)

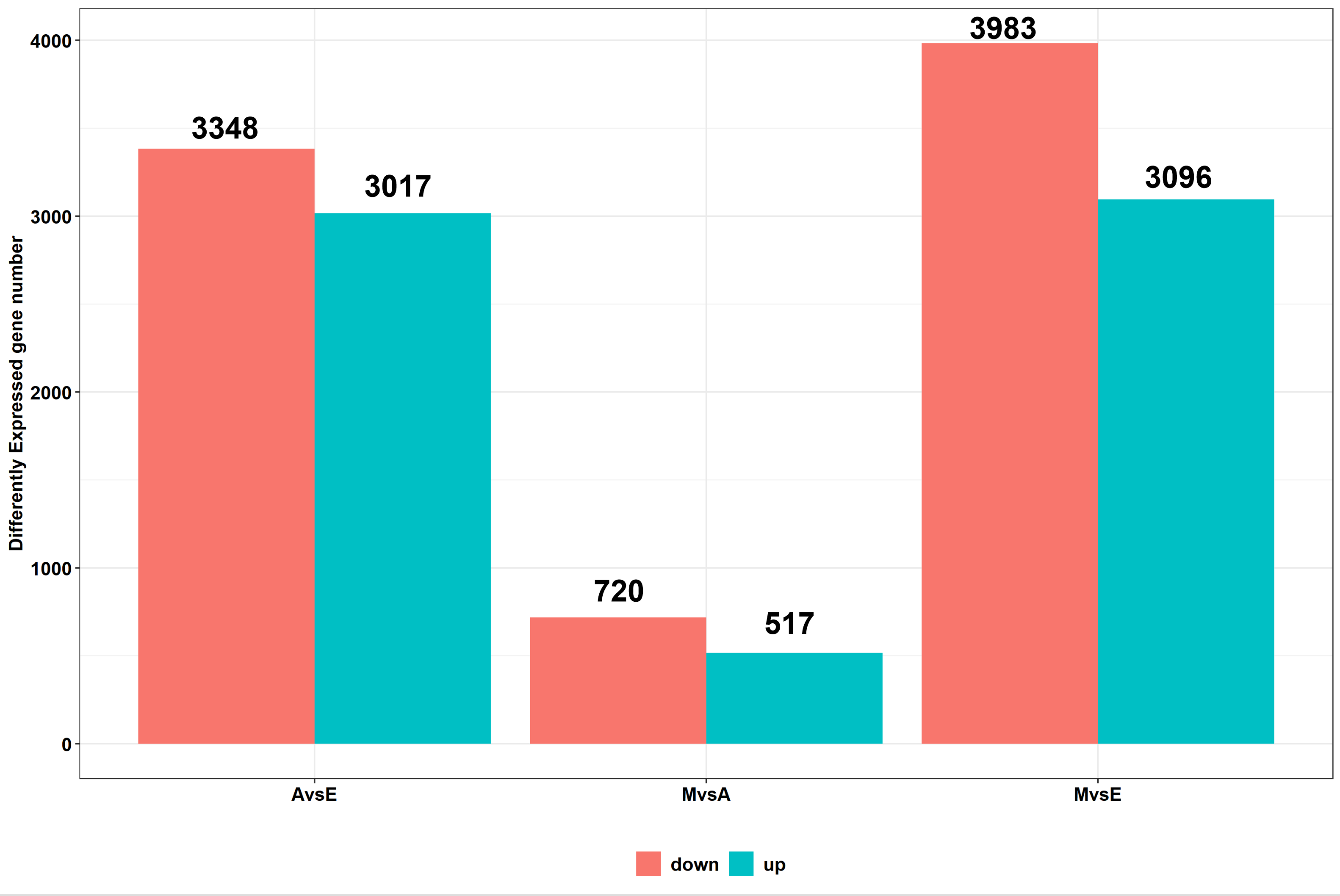

Supplement: Supplementary file 1 [file genes-10-00970-s001.zip › Figure s1.png]

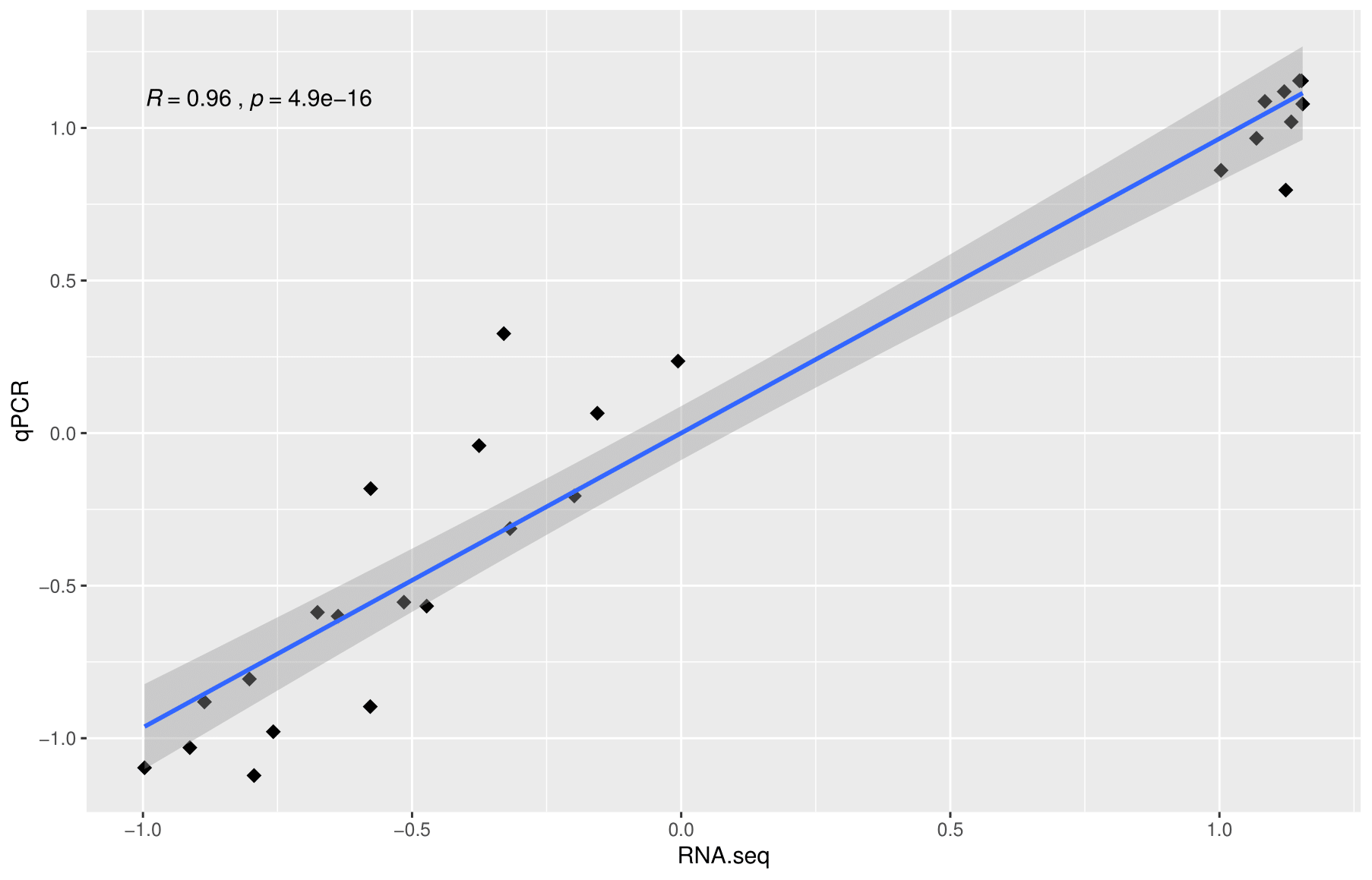

Supplement: Supplementary file 1 [file genes-10-00970-s001.zip › Figure S2.png]
